# 肺癌循环肿瘤细胞临床应用的研究进展

**DOI:** 10.3779/j.issn.1009-3419.2011.06.12

**Published:** 2011-06-20

**Authors:** 伟 平, 向宁 付

**Affiliations:** 430030 武汉，华中科技大学同济医学院附属同济医院普胸外科 Department of General Toracic Surgery, Tongji Hospital, Tongji Medical College, Huazhong University of Science and Technology, Wuhan 430030, China

肺癌的发病率和死亡率在恶性肿瘤中居首位，肿瘤复发或远处转移是导致患者死亡的主要原因。循环肿瘤细胞（circulating tumor cell, CTC）在肺癌的血行转移中具有极其重要的作用。临床研究^[1-5]^显示CTC与肺癌临床分期有关，在肺癌的早期诊断、疗效监测和预后评估及个体化治疗中具有重要的临床意义。

## 1 肺癌CTC概述

肺癌是一种全身性疾病，其发病率正逐年上升，全球每年死于肺癌的人数大约有110万。很多患者确诊时已为晚期，错过最佳手术时期，此外无法耐受放疗或化疗及化疗后耐药等原因导致肺癌5年生存率不到15%。Ⅰ期经手术根治性治疗的非小细胞肺癌（non-small cell lung can-cer, NSCLC）仍有超过30%患者在5年内复发^[6]^，且复发部位多为全身性转移，提示现有临床分期的早期就可能已有肺癌微转移的发生^[7]^。血行播散是肺癌转移的重要途径，肿瘤细胞可以通过血液循环发生远处转移。肿瘤细胞进入血液循环是肿瘤发生远处转移的关键步骤之一，因此对肺癌CTC的检测研究受到越来越多的重视。

CTC目前定义为自发或因诊疗操作由实体瘤或转移灶释放进入外周血循环的肿瘤细胞^[8]^。源于肺癌实体瘤的肿瘤细胞不断增殖，通过上皮间质转化（epithelial-mesenchymal transition, EMT）等过程在分子转录水平、蛋白质修饰和细胞表型上的改变而变得具有侵袭性，从而利于逃离组织束缚进入循环系统，成为具有侵袭转移能力的CTC^[9]^。研究^[1-5]^发现检测肺癌CTC有助于早期诊断及判断患者预后，与临床分期有关，在治疗期间定期检测并监测CTC的变化，可为评价治疗方案的敏感性提供依据，有利于开展个体化的治疗。

## 2 肺癌CTC的检测

现有分离、检测肺癌CTC的技术方法较多且各有利弊。逆转录聚合酶链反应（reverse transcriptase-polymerase chain reaction, RT-PCR）及其各种改进技术目前在肺癌CTC的检测中应用最广泛。RT-PCR检测CTC是在PCR的基础上扩增由肿瘤特异性mRNA序列逆转录的DNA片段，从而识别组织或肿瘤特异性mRNA的表达或某些基因改变后RNA水平的异常。由于这些特异性mRNA半衰期较短，在细胞死亡后会被RNA酶迅速降解，且通常不表达于正常外周血细胞中，所以在肺癌患者外周血中检测到特异性mRNA可间接提示CTC的存在^[10]^。近些年新开发的荧光定量RT-PCR、巢式RT-PCR及实时定量RT-PCR，虽提高了检测的敏感性和特异性，但仍存在CTC计数障碍^[11]^，而Cell Search System^[12]^和CTC-chip^[3]^等新技术的应用，可进行肺癌CTC计数，便于实时监测，且不影响细胞活性，有利于下一步的分子生物学和遗传学分析。

Cell Search System是基于免疫学的检测方法，CTC用EpCAM抗体磁珠进行富集后，在一个外加强力磁场的作用下可从血液中被提取，对分离出来的CTC进行通透、固定，用DAPI荧光核染料、CD45荧光抗体和CK8、CK18以及CK19荧光抗体标记细胞，然后采用半自动四色荧光显微镜Cell-Spotter Analyzer检测分析，符合肿瘤细胞学形态特征并且CK-PE+、DAPI+和CD45-的细胞被定义为CTC。此系统能从7.5 mL的全血中检测到单一的肿瘤细胞。CTC-chip是近年来新出现的一种基于微流体学的检测技术。它在一张与标准载玻片尺寸相同的硅片上面覆盖8万个显微位点，每一个位点都包被上能够捕捉CTC的抗体。当血液样品通过微流芯片时，这些位点丛即可确保CTC在流过芯片前被捕获。

## 3 肺癌CTC与早期诊断

肺癌的早期诊断对治疗效果至关重要，早期发现及时治疗是提高生存率的关键。研究^[2, 13]^显示CTC对肺癌早期诊断具有潜在的应用价值。目前，在无明显临床症状和转移灶的Ⅰ期患者体内也可检测到CTC，说明现有临床分期的早期就已存在肺癌细胞的播散^[7]^，并能在大量聚集后形成具有高度转移潜能的循环肿瘤微栓（circulating tumor microemboli, CTM）。理论上使用基于肿瘤细胞大小的分离法（isolation by size of epithelial tumor cells, ISET）可对CTM进行富集^[11]^，这使未来对高危患者早期筛查协助诊治成为可能。且目前已有学者借助于CTC对转移性小细胞肺癌（small cell lung cancer, SCLC）早期做出诊断，Bevilacqua等^[2]^最初对1例患者肺组织穿刺活检诊断为低分化神经内分泌瘤，但应用Cell Search System在外周血中检测到EpCAM和CK阳性的CTC，然而EpCAM通常不表达于低分化神经内分泌瘤，其后对肝脏穿刺活检发现肝内肺转移灶，最后确诊为EpCAM和CK阳性的SCLC。与目前的影像学、肿瘤标志物等相比，CTC在肺癌的早期诊断应用中尚处于起步阶段，检测CTC能否作为早期诊断的指标，还需进一步探讨，但联合多项检查后的综合判断会提高临床医师诊疗的警惕性。

## 4 肺癌CTC与临床分期

目前临床上广泛使用的肺癌分期方法仍以解剖学特征为基础，但随着分子机制在肿瘤生物学上的发展，近年来已有不少学者提出分子分期的概念^[12]^。有研究^[13]^发现直径≤1 cm的肿瘤就已发生转移，无临床转移病灶的Ⅰ期肺癌中也可检测到CTC^[7]^。虽然血液中检测到CTC并不意味着一定存在转移灶，但研究^[1, 7, 14]^显示肺癌CTC与肿瘤分期具有明显的相关性。Kurusu等^[7]^应用RT-PCR检测103例手术治疗的NSCLC患者外周血中的肿瘤组织特异性CEA mRNA，发现阳性率很大程度上与术前术后的临床分期有关。Yie等^[14]^应用RT-PCR ELISA对143例NSCLC患者进行检测，发现表达Survivin mRNA的CTC与肿瘤侵犯程度和淋巴结转移状态有关。Sher等^[1]^在其研究中也发现晚期肺癌患者CTC阳性率明显高于早期患者，且CTC可作为TNM分期系统的有益补充。虽然尚不能将CTC迅速应用于仍以解剖学特征为基础的肺癌分期系统，但以上研究提示CTC有潜力协助分期，结合影像学或病理学的检查结果，更加综合的反应患者当期病情。目前，尽管在外周血中检测到CTC并不意味着体内一定存在微转移灶，但有学者发现CTC在肿瘤的演变过程中存在进化，从原发部位向远处器官扩散时会出现基因型的改变，可成为肿瘤原发部位和微转移灶之间的桥梁，并能反应微转移灶的性质^[5, 15]^。这可弥补影像学和肿瘤标志物等检查无法及时精确反映转移灶性质的缺陷，对病情分期的评估和治疗方案的制定都具有影响，从而选择适宜的治疗方式，避免过多的细胞毒性及治疗方案的不足，在提高TNM分期准确性的同时对肺癌个体化治疗的开展也具有一定的意义。

## 5 肺癌CTC与预后

对肺癌CTC的研究^[4, 15, 16]^显示CTC具有重要的预后价值。Chen等^[16]^通过巢式RT-PCR法检测行放化疗的67例NSCLC患者，发现治疗前CK19 mRNA阴性而治疗后转为阳性的患者的无进展生存期（progression free survival, PFS）和总生存期（overall survival, OS）均较短；在治疗过程中CK19 mRNA持续阳性的患者预后最差。多变量分析显示CK19 mRNA可作为NSCLC患者PFS和OS的独立预测因子。Yoon等^[17]^应用巢式实时RT-PCR检测79例NSCLC患者的TTF-1 mRNA，发现术后阳性患者的PFS比阴性患者较短，提示TTF-1 mRNA阳性的CTC与病情进展程度有关。Tanaka等^[4]^应用Cell Search System发现CTC数量在肺癌进展和转移过程中增加，对监测病情变化和判断预后具有重要意义。目前，已有大量研究显示晚期肺癌患者CTC阳性率明显高于早期患者，但CTC与肺癌预后的研究大都尚限于NSCLC，针对SCLC的研究较少，且肺癌CTC的检测方法和使用的肿瘤标志物亦不同，所以关于CTC与肺癌的预后关系尚需病例数更多、设计更为严格的多中心临床研究进一步证实。

## 6 肺癌CTC与治疗

当前对肺癌的治疗是基于肿瘤病理分级、患者一般状况及肿瘤发展趋势等因素之上的综合治疗。但遵循统一的诊断标准建立的治疗方案缺乏个体化。随着对CTC研究的深入发现其可反映肿瘤组织基因的表达变化，有助于监测疗效及建立个体化的治疗方案。

肺癌肿瘤组织基因的表达或突变可在治疗过程中变化，从而导致药物敏感性的改变，如耐药基因T790M可导致对吉非替尼敏感的NSCLC治疗失败。Maheswaran等^[5]^应用CTC-chip检测接受吉非替尼治疗的NSCLC患者的CTC，发现CTC的基因突变与肿瘤组织具有高度一致性，同样存在*EGFR*突变（T790M），且在治疗过程中监测发现CTC数量的改变可以反映患者病情的变化趋势和治疗的疗效。在这之前，Nagrath等^[3]^对9例NSCLC患者的研究结果与之相似，发现在评估化疗的疗效上CTC数量的改变与按照实体瘤的疗效评价标准（Response Evalua-tion Criteria in Solid Tumors, RECIST）进行CT影像学检查的结果具有高度一致性。Bepler等^[18]^对30例接受吉西他滨方案治疗的局部进展期NSCLC患者跟踪随访，发现RRM1 mRNA的表达与疗效呈负相关，即吉西他滨对高表达者疗效差，对低表达者疗效好。Wu等^[19]^应用Cell Search System对12例化疗2个疗程的肺癌患者跟踪随访，也发现CTC数量与化疗后影像学的应答存在关系，提示CTC可实时监测化疗肺癌患者的疗效。

总之，CTC对肺癌个体化治疗有潜在的重大意义，有望依靠非侵入性的血液检测获悉当前治疗的疗效，起到实时监测的作用，从而利于临床医师及时调整治疗策略，达到个体化治疗目的，但能否替代原发组织标本指导分子靶向治疗，并对服用吉非替尼等分子靶向药物治疗进行实时监测及避免耐药基因的出现而影响疗效，尚需通过大规模的前瞻性研究来证实。

## 7 展望与结语

伴随现代生物学技术的发展，CTC检测的敏感性和特异性均明显提高，使探讨其临床应用价值成为可能。2004年Cell Search System已被美国食品药物管理署（FDA）批准进入临床，但只限于预测转移性乳腺癌（metastatic breast cancer, MBC）、前列腺癌和结直肠癌的PFS和OS的应用，而未能临床应用于肺癌。对于MBC、前列腺癌和结肠癌而言，CTC已被认为是一种可靠的预后指标，能在治疗过程中预测病情的进展和患者的生存时间；而监测结果能更早地为临床医师提供更丰富的信息，从而有利于制定更明确的治疗方案。37%MBC、57%前列腺癌、30%结肠癌患者外周血样本中都可以检出≥2个CTC，而健康志愿者和非恶性肿瘤患者外周血样本中几乎没有这种现象^[20]^。

2007年Nagrath等^[3]^率先报道了CTC-chip比Cell Search System分离纯度高出两个数量级，更有利于分子生物学检测。为能将CTC用于肺癌的日常临床工作，尚需通过大规模、多中心的研究建立快速、灵敏、精确的检测方法。目前，肺癌CTC的检测及临床应用还没有一致的标准，但从以上理论和已有的研究结果来看，CTC至少具有以下几点潜在应用价值，即在早期患者中发现隐匿性微转移、监测肺癌患者病情进展和评价患者对治疗的反应。

2009年美国临床肿瘤协会（ASCO）大会上就有学者高度强调CTC在癌症患者中的重要应用价值，认为CTC可作为实时液体活检和临床常规检测中较为理想的标本来源。对肺癌CTC的进一步研究将有助于阐明肺癌的生物学特性，在肺癌未来的综合诊治中可能将更多的考虑CTC的存在及其特性，相应检测有望成为肺癌患者的临床常规检查，为患者带来福音。
